# Clinicopathological characteristics of *ROS1-* and *RET-*rearranged NSCLC in caucasian patients: Data from a cohort of 713 non-squamous NSCLC lacking KRAS/EGFR/HER2/BRAF/PIK3CA/ALK alterations

**DOI:** 10.18632/oncotarget.18408

**Published:** 2017-06-08

**Authors:** Frédéric Dugay, Francisco Llamas-Gutierrez, Marjory Gournay, Sarah Medane, François Mazet, Dan Christian Chiforeanu, Emmanuelle Becker, Régine Lamy, Hervé Léna, Nathalie Rioux-Leclercq, Marc-Antoine Belaud-Rotureau, Florian Cabillic

**Affiliations:** ^1^ Department of Cytogenetics and Cell Biology, CHU de Rennes, Rennes, France; ^2^ IRSET UMR INSERM 1085, Faculté de Médecine, Université de Rennes 1, Rennes, France; ^3^ Department of Pathology, CHU de Rennes, Rennes, France; ^4^ Department of Pneumology, CHU de Lorient, Lorient, France; ^5^ Department of Pneumology, CHU de Rennes, Rennes, France; ^6^ INSERM, INRA, Université de Rennes 1, Université Bretagne Loire, Nutrition Metabolisms and Cancer, Rennes, France

**Keywords:** non-small cell lung cancer, ROS1, RET, fusion genes, caucasian population

## Abstract

Targeted therapies have substantially changed the management of non-small cell lung cancer (NSCLC) patients with driver oncogenes. Given the high frequency, *EGFR* and *ALK* aberrations were the first to be detected and paved the way for tyrosine kinase inhibitor (TKI) treatments. Other kinases such as *ROS1* and more recently *RET* have emerged as promising targets, and ROS1 and RET TKIs are already available for precision medicine.

We screened a large cohort of 713 Caucasian non-squamous NSCLC patients lacking *EGFR*/*KRAS*/*BRAF*/*HER2*/*PI3KCA*/*ALK* aberrations for *ROS1* and *RET* rearrangements using fluorescence *in situ* hybridization to determine the frequency and clinicopathological characteristics of ROS1- and RET-positive patients.

Frequencies of *ROS1* and *RET* rearrangements were 2.1% and 2.52%, respectively. Contrary to common belief, both *ROS1* and *RET* rearrangements were detected in patients with a history of smoking, and the *RET*-positive patients were not younger than the negative patients. Moreover, *RET* but not *ROS1* rearrangement was associated with the female gender. Nearly half of the *ROS1*-rearranged patients were successfully treated with ROS1 TKIs. In contrast, only 5/18 RET-positive patients received off-label RET TKIs. Two patients had stable disease, and three experienced disease progression. In addition to the 18 RET-positive cases, 10 showed isolated 5′ signals. The clinical relevance is unknown but if the frequency is confirmed by other groups, the question whether these patients are eligible to TKIs will arise. More potent RET TKIs are under development and may improve the response rate in RET-positive patients. Therefore, we recommend the routine implementation of RET testing in non-squamous NSCLC patients, including those with a history of smoking.

## INTRODUCTION

Non-small cell lung cancer (NSCLC) is one of the leading causes of cancer-related death worldwide, accounting for approximately 1.8 million deaths every year [1]. During the past few years, several gene aberrations have been identified as oncogenic drivers in NSCLC, including epidermal growth factor receptor (*EGFR*) and Kirsten rat sarcoma viral oncogene homolog (*KRAS*) [2]. In 2007, anaplastic lymphoma kinase (*ALK*) was the first gene reported to cause lung adenocarcinoma upon a chromosomal rearrangement resulting in a fusion gene [3]. Very soon, impressive clinical responses with ALK tyrosine kinase inhibitor (TKI) crizotinib were reported [4] and prompted further investigation on the role of fusion genes in NSCLC. Those efforts led to the identification of new fusion genes involving *ROS1* and rearranged during transfection (*RET*) [5, 6]. Both ROS1 and RET belong to the receptor tyrosine kinase superfamily. ROS1 belongs to the insulin subgroup and shares a high degree of homology within the kinase domain with ALK. Its precise role still remains to be established [7]. RET is phylogenetically related to the fibroblast growth factor receptors and is required for the development of the kidneys and nervous system as well as for the maturation and maintenance of hematopoietic stem cells [8]. The oncogenic role of *ROS1* was described in 1987 [9], but the role of ROS1 fusion proteins in NSCLC was demonstrated later with the identification of SLC34A2-ROS1 fusion in 2007 [5]. Likewise, aberration in *RET* has long been known to be involved in thyroid cancers, but the first *RET* fusion gene in NSCLC was found in early 2012 [6]. Since then, various ROS1 and RET fusion-partners have been reported in NSCLC, and all together these variants are thought to cause 2% to 4% of lung adenocarcinoma [10]. *ROS1*- and *RET*-rearranged NSCLC patients share clinical characteristics with *ALK*-positive NSCLC patients, including young age of onset and minimal to no previous history of smoking [11, 12]. Interestingly, ROS1- and RET-targeted therapies are already available for precision medicine.

Here, we report the frequency of *ROS1* and *RET* rearrangements in a large cohort of 713 Caucasian patients with non-squamous NSCLC lacking EGFR/KRAS/BRAF/HER2/PI3KCA/ALK alterations by using fluorescence *in situ* hybridization (FISH). We investigate the correlation between fusion-positive tumors and clinicopathological features, and we report the clinical outcome of patients treated with crizotinib or investigational RET TKIs.

## RESULTS

### Percentage of samples with *RET* rearrangement slightly exceeds that of *ROS1*

*ROS1* rearrangements were found in 15 (2.1%) samples (Table 1). The hybridization profiles were as follows: isolated 3′ signals (n=4), split signals (n=7) or a combination of both (n=4) (Table 2, Figure 2B and 2D). ROS1 protein was detected in 13/15 *ROS1*-rearranged samples. In addition, *RET* rearrangements were found in 18 (2.5%) samples: most of them (n=14) had split signals whereas 4 samples exhibited isolated 3′ signals (Table 3, Figure 2F and 2H). Ten additional cases showed isolated 5′ signals and were considered RET-negative (Figure 3D). The copy number of genes was also recorded in both rearranged and non-rearranged cases (Table 4). Of the samples, 7% had a single copy of *ROS1*, and one sample exhibited *ROS1* amplification without protein overexpression (Figure 3A and 3B). Copy number gain (CNG) was more frequently observed for *RET* compared to *ROS1* (Table 4). No significant differences in CNG frequencies were noted between rearranged and non-rearranged samples.

**Table 1:** Clinicopathological characteristics of *ROS1*- and *RET*-rearranged patients

	ROS1			RET		
	+	-	p	+	-	p
**Number(n=713)**						
	15	698		18	695	
**Age (years)**						
Mean	59.1	65.6	**0.02**	67.7	65.4	0.55
SD	14	10.7		15.4	10.7	
**Sex**						
Male	9	479	0.58	8	480	**0.04**
Female	6	219		10	215	
**Smoking history (n=551)**						
Never	6	93	**0.03**	7	92	**0.02**
Current/former	8	444		10	442	
**Stage at diagnosis (n=574)**						
I/II	2	126	0.64	5	123	0.09
III	5	148		1	152	
IV	7	286		12	281	
**Tumor histology**						
Adenocarcinoma	14	587	0.49	17	584	0.33
Other	1	111		1	111	
**Adenocarcinoma subtype**						
Solid	4	182	0.73	9	177	0.27
Acinar	5	157		4	158	
Lepidic	3	85		1	87	
Papillary	1	111		1	111	
Undetermined/cytology	1	52		2	51	

**Figure 1:**
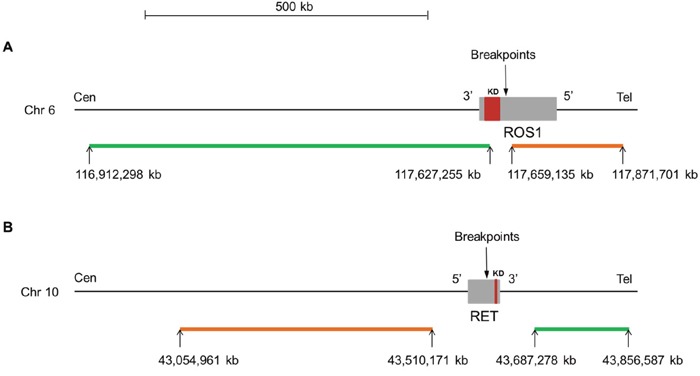
Map for *ROS1* **(A)** and *RET* **(B)** dual color break-apart probes (ZytoLight^®^ SPEC ROS1 and RET, ZytoVision). For both genes, the orange and green fluorochrome direct labeled probes hybridize proximal (5′ end) and distal (3′ end) to the genes, respectively. The known breakpoints in *ROS1* are located in introns 31, 33 and 34 and are proximal to the kinase domain (exons 36 to 41). For *RET*, the known breakpoints are located in introns 10 and 11 and are proximal to the kinase domain (exons 12 to 19). The precise location of the genes on the chromosome, the size (kb) and orientation of the genes are indicated according to the Human assembly GRCh37/hg19. kb: kilobase pair; Cen: centromere; Tel: telomere; Chr: chromosome; KD: kinase domain.

**Figure 2:**
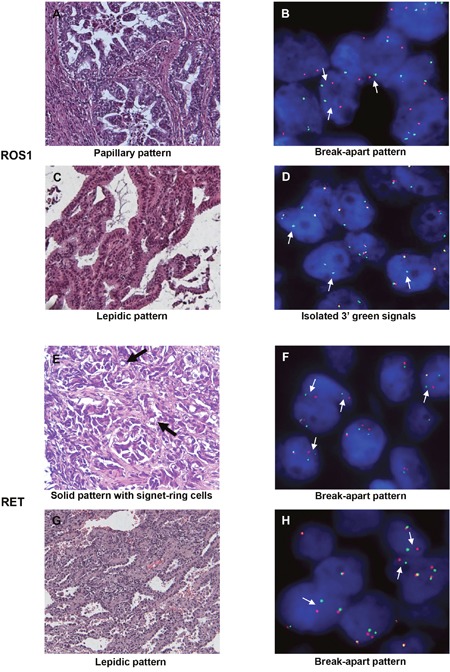
Images of *ROS1*- or *RET*-rearranged lung adenocarcinoma H&E staining **(A, C, E, G)** and corresponding FISH profiles **(B, D, F, H)** are shown. Thin white arrows indicate split signals or isolated 3′ green signals. Thick black arrows indicate signet-ring cells. H&E: hematoxylin and eosin (200x); FISH: fluorescence *in situ* hybridization.

**Figure 3:**
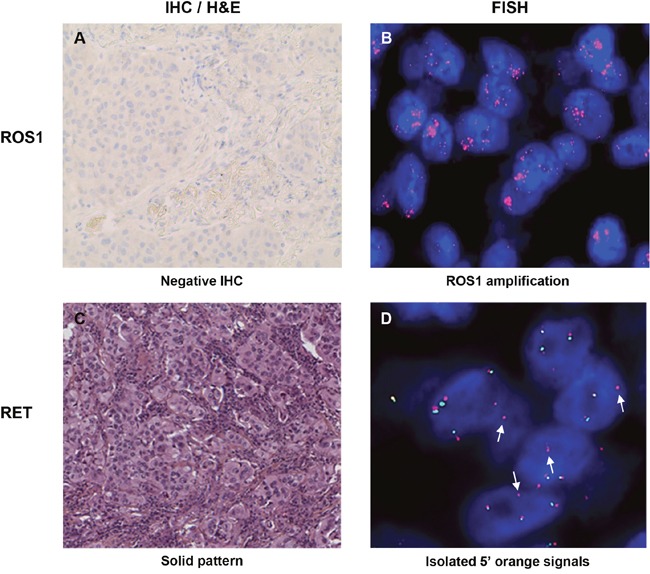
Images of samples with *ROS1* amplification **(A-B)** and atypical isolated 5′ signal pattern for *RET* **(C-D)**. White arrows indicate isolated 5′ orange signals. IHC: immunohistochemistry; H&E: hematoxylin and eosin (200x); FISH: fluorescence *in situ* hybridization.

**Table 2:** Clinical, histological and molecular features of *ROS1*-rearranged patients

N°	Sex / Age	ROS1 diagnosis mm/yy	Last news or death (ς) mm/yy	Smoker (pack-year)	TNM	Stage	Histological subtype / Predominant pattern	FISH-positive rate (%)	FISH pattern	Lines of treatment Date mm/yy (nb of cycles) - Cause of therapeutic escape	Best response
1	M/49	02/14	01/16	Current (10)	T1bN2M0	IIIa	ADK / Acinar signet-ring cells	90	IGS	1. Surgery + adjuvant cisplatin/vinorelbine	Complete response
2	F/51	04/14	11/15 (ς)	Former (10)	T3N2M1b	IV	ADK / Acinar	90	Split	1. Cisplatin/pemetrexed 04/14 (x1) - Stop for toxicity	Not evaluated
										2. Crizotinib from 05/14 to 07/15 - Liver metastases	Partial response
										3. Ceritinib from 08/15 to 09/15	Progression of disease
										4. Nivolumab 10/15	Progression of disease
3	F/57	04/14	06/16	Never	T4N1M0	IIIa	ADK / Lepidic	50	Split	1. Surgery + adjuvant cisplatin/pemetrexed	Complete response
4	M/56	12/14	01/17	Current (60)	T4Nx	IIIa	ADK / Solid	60	IGS	1. Surgery + adjuvant cisplatin/pemetrexed	Partial response
										2. Pemetrexed maintenance from 07/15 - ongoing	Stable disease
5	F/90	01/15	NA	NA	NA	NA	ADK / Solid	80	IGS+split	Best supportive care	/
6	M/49	02/15	01/17	Former (25)	T4N3M1	IV	ADK / Solid	50	IGS+split	1. Cisplatin/pemetrexed/bevacizumab from 12/12 to 01/13 (x2)	Progression of disease
										2. Docetaxel from 02/13 to 05/13 (x6), then therapeutic break	Stable disease
										3. Paclitaxel from 02/14 to 05/14 (x3)	Partial response
										4. Erlotinib from 08/14 to 10/14	Progression of disease
										5. Gemcitabine from 10/14 to 12/14 (x3)	Progression of disease
										6. Docetaxel from 01/15 to 02/15 (x3)	Progression of disease
										7. Crizotinib from 03/15 to 09/16 - Brain metastases	Partial response
										8. Ceritinib from 09/16 to 01/17 - Pericardial metastases	Partial response
7	M/30	04/15	02/17	Never	T2aN3M0	IIIb	ADK / Lepidic	70	IGS+split	1. Carboplatin/paclitaxel from 03/15 to 09/15	Partial response
										2. Crizotinib from 10/15 to 03/16, then therapeutic break - brain metastasis 08/16	Stable disease
										3. Carboplatin/paclitaxel from 09/16 to 10/16	Progression of disease
										4. Lorlatinib 01/17 - Stop for toxicity (intersitial pneumopathy)	Not evaluated
8	M/52	08/15	07/15 (ς)	Never	T4NxM1b	IV	Large cell carcinoma	90	IGS+split	1. Carboplatin/paclitaxel (x1)	Progression of disease
9	F/59	09/15	07/16 (ς)	Current (80)	T3N3M1b	IV	ADK / Solid	90	Split	1. Surgery + adjuvant carboplatin/pemetrexed	Partial response
										2. Pemetrexed maintenance from /01/16 to 03/16	Stable disease
										3. Crizotinib from 05/16 to 07/16*	Progression of disease
10	M/77	02/16	01/17	Former (5)	T2N2M0	IIIa	ADK / Papillary	90	Split	1. Surgery + adjuvant carboplatin/vinorelbine	Progression of disease
										2. Crizotinib from 11/16 - ongoing	Complete response
11	M/67	02/16	03/16	Current (50)	T2N0M0	Ib	ADK / Acinar	90	Split	1. Surgery	Complete response
12	M/67	05/16	01/17	Former (20)	T1bN3M1a	IV	ADK / Acinar	90	Split	1. Carboplatin/pemetrexed/bevacizumab from 02/16 to 05/16 (x4)	Stable disease
13	M/71	05/16	06/16	Never	T1aN0	Ia	ADK / Acinar	70	IGS	1. Surgery	Complete response
14	F/58	06/16	02/17	Never	T2aN3M1b	IV	ADK / Cytology	80	Split	1. Crizotinib from 08/16 - ongoing	Partial response
15	F/54	06/16	02/17	Never	T4N0M1	IV	ADK / Lepidic	70	IGS	1. Cisplatin/pemetrexed	Progression of disease
										2. Erlotinib	Partial response
										3. Crizotinib	Complete response

M: Male; F: Female; Split: Split signals; IGS: Isolated 3′ Green Signal; NA: Not Available; * brain metastasis at the time of diagnosis

**Table 3:** Clinical, histological and molecular features of *RET*-rearranged patients

N°	Sex / Age	RET diagnosis mm/yy	Last news or death (ς) mm/yy	Smoker (pack-year)	TNM	Stage	Histological subtype / Predominant pattern	FISH-positive rate (%)	FISH pattern	Line of treatment Date mm/yy (nb of cycles) - Cause of therapeutic escape	Best response
1	M/75	02/14	01/16	Former (10)	T1aN0M0	Ia	ADK / Acinar	70	Split	1. Surgery	Complete response
2	F/54	05/14	09/14 (ς)	Never	T4N3M1b	IV	ADK / Solid	70	Split	1. Brain radiotherapy + cisplatine/pemetrexed from 05/14 to 07/14	Progression of disease
										2. Vandetanib from 08/14 to 09/14	Progression of disease
3	M/60	08/14	01/16 (ς)	Current (35)	T4N3M1	IV	ADK / Papillary	50	Split	1. Cisplatin/pemetrexed/bevacizumab from 08/14 to 10/14	Partial response
										2. Nivolumab from 09/15 to 12/15	Progression of disease
										3. Paclitaxel from 12/15 to 01/16	Progression of disease
4	F/94	09/14	09/15	Former (30)	T1N0M0	Ia	ADK / Solid	50	Split	Best supportive care	/
5	F/77	10/14	12/16	Never	TxNxM1	IV	ADK / Acinar	70	IGS	1. Carboplatin/pemetrexed from 08/14 to 11/14 (x4)	Partial response
										2. Pemetrexed maintenance from 12/14 to 04/15	Progression of disease
										3. Sunitinib from 05/15 to 07/15	Progression of disease
										4. Paclitaxel from 09/15 to 10/16, then therapeutic break	Stable disease
6	F/87	10/14	01/15 (ς)	Former (40)	T4N2M1	IV	ADK / Solid signet-ring cells	50	Split	Best supportive care	/
7	M/59	10/14	02/17	Never	T4N2M1	IV	NOS	70	Split	1. Cisplatin/pemetrexed/bevacizumab from 8/14 to 10/14 (x4)	Stable disease
										2. Pemetrexed maintenance 11/14 - Stop for toxicity, then therapeutic break	Stable disease
										3. Erlotinib from 08/15 to 02/16	Progression of disease
										4. Nivolumab from 03/16 to 06/16	Progresion of disease
										5. Vandetanib from 12/16 - ongoing	Stable disease
8	F/75	04/15	03/16	Never	T2N0M0	Ib	ADK / Cytology	80	Split	1. Surgery	Complete response
9	M/50	05/15	02/17 (ς)	Current (20)	T1N3M1	IV	ADK / Solid	80	Split	1. Cisplatin/pemetrexed from 08/13 to 10/13 (x4)	Partial response
										2. Pemetrexed maintenance from 11/13 to 08/14, then therapeutic break	Stable disease
										3. Pemetrexed at bone progression from 09/15 to 03/16 - Stop for toxicity	Stable disease
										4. Nivolumab from 10/16 to 01/17	Progression of disease
10	F/44	06/15	04/16 (ς)	Never	T1N3M1b	IV	ADK / Solid	50	Split	1. Cisplatin/pemetrexed/bevacizumab from 06/15 to 09/15	Partial response
										2. Paclitaxel from 10/15 to 03/16	Partial response
11	F/83	06/15	06/15 (ς)	Never	T3N3M1	IV	ADK / Solid	60	Split	1. Gefitinib from 28/05/15 to death (3 days)	Progression of disease
12	F/88	07/15	07/15 (ς)	NA	M1a	IV	ADK / Undetermined	50	Split	1. Gefitinib from 06/15 to death	Progression of disease
13	M/63	11/15	12/15	Current (40)	T2N0M0	Ib	ADK / Solid	50	IGS	1. Surgery	Complete response
14	M/57	12/15	01/17 (ς)	Current (70)	T4N3M1	IV	ADK / Acinar	60	IGS	1. Cisplatin/docetaxel from 06/14 to 08/14 (x4)	Partial response
										2. Pemetrexed from 09/14 to 06/15 - Stop for toxicity	Stable disease
										3. Nivolumab from 11/15 to 12/15	Progression of disease
										4. Docetaxel from 01/16 to 05/16, then therapeutic break - Brain metastases	Partial response
										5. Erlotinib 08/16	Progression of disease
										6. Sunitinib 11/16	Progression of disease
15	M/68	12/15	12/15 (ς)	Former (5)	M1	IV	ADK / Solid signet-ring cells	60	Split	1. Cisplatin/pemetrexed 12/15 (x1)	Progression of disease
16	F/80	01/16	03/17	Never	T2N2M1	IV	ADK / Solid signet-ring cells	90	Split	1. Carboplatin/paclitaxel from 06/16 to 05/16 (x6)	Stable disease
										2. Paclitaxel maintenance (x9) - Stop for toxicity 11/16	Stable disease
										3. Vandetanib from 12/16 - ongoing	Stable disease
17	M/61	04/16	12/16	Current (60)	T4N2M0	IIIb	ADK / Acinar	80	IGS	1. Radiotherapy + carboplatin/paclitaxel from 03/16 to 05/16	Progression of disease
										2. Pemetrexed from 09/16 to 12/16 (x5)	Stable disease
18	F/43	06/16	07/16	Former (10)	T1aN0	Ia	ADK / Lepidic	90	Split	1. Surgery	Complete response

M: Male; F: Female; NOS: not otherwise specified; Split: Split signals; IGS: Isolated 3′ Green Signal; NA: Not Available

**Table 4:** *ROS1* and *RET* gene copy number in rearranged and non-rearranged samples

		1 copy	2 copies	3 to 6 copies	7 to 10 copies	> 10 copies or clusters
**ROS1**	-	49 (7%)	307 (43.9%)	341 (49%)	0	1 (0.1%)
	+	1 (6.7%)	9 (60%)	5 (33.3%)	0	0
**RET**	-	10 (1.4%)	220 (31.6%)	441 (63.5%)	24 (3.5%)	0
	+	0	6 (33.3%)	11 (61.1%)	1 (5.5%)	0

### *RET* rearrangement is more frequent in women and can cause NSCLC in smokers

*ROS1* rearrangement was more frequent in younger patients (p=0.02) but was not found to be significantly associated with gender (Tables 1 and 2). Six patients were never smokers and 8 patients were former or current smokers (Tables 1 and 2). Most of the *ROS1* rearrangements were detected in adenocarcinoma (14/15) but no enrichment in a particular histological subtype was demonstrated (Tables 1 and 2). *ROS1* rearrangement was found in acinar (n=5), solid (n=4), lepidic (n=3) and papillary (n=1) adenocarcinoma (Tables 1 and 2, Figure 2A and 2C).

*RET* rearrangement was not significantly associated with age but was more frequently found in female patients (p=0.04) (Tables 1 and 3). Seven patients were never smokers and 10 patients were former or current smokers (Tables 1 and 3). Most of the RET-positive cases were detected in advanced stage tumors (13/18 stages III/IV). *RET* rearrangement was found in 17 adenocarcinoma and in one carcinoma not otherwise specified (NOS). Solid growth pattern tended to be more frequent (9/17), and signet-ring cells were reported in three samples (Tables 1 and 3, Figure 2E and 2G).

### Unlike ROS1, therapy for RET-positive patients still needs to be refined

The treatments given and disease outcomes were collected. Among the ROS1-positive patients, 7/15 received ROS1-targeted therapy. Five patients achieved partial (n=3) or complete (n=2) responses. One patient had stable disease and one patient who developed metastases prior to starting crizotinib experienced progressive disease (Table 2). After initial crizotinib relapse, 3 patients were given second- or third-generation TKIs (ceritinib or lorlatinib). In addition, 4 patients underwent curative surgery, and 3 patients were treated with chemotherapy alone. Among the RET-positive patients, only 5/18 received the RET inhibitors vandetanib or sunitinib. Among them, 2 patients had stable disease and three experienced clinical deterioration with disease progression (Table 3). In addition, 4 patients benefited from curative surgery and 5 patients were treated with chemotherapy alone. Of note, one ROS1-positive and 4 RET-positive patients were given the anti-PD-1 immune checkpoint inhibitor nivolumab, but none experienced clinical response.

## DISCUSSION

Most of the available data regarding gene rearrangement in NSCLC, especially *RET* fusion, come from Asian patients. Although to a lesser extent than in EGFR-driven tumors, ethnic heterogeneity have been reported in fusion gene-induced tumors [2, 13, 14]. Hence, our study provides specific information about the Caucasian population. Among 713 non-squamous NSCLC lacking EGFR/KRAS/HER2/BRAF/PI3KCA/ALK aberrations, we reported 2.1% and 2.5% *ROS1* and *RET* rearrangements, respectively. As the cohort composition is highly variable across studies (NSCLC, non-squamous NSCLC, ADK or ADK lacking mutations), interstudy comparison requires prior adjustments. The levels found in this study were lower than those reported in the Asian population [15–17], but they were in line, once adjusted, with European studies [18–20]. Indeed, extrapolation to unselected NSCLC patients would give between 0.5% to 1% ROS1-positive cases i.e. similar levels than those reported by Warth et al. (0.6%, 9/1478) and Jurmeister et al. (0,8%, 4/473) [18, 19]. The notion that oncogenic fusions mainly affect young, female and non-smoker patients originated from the knowledge gained from studying ALK. However, recent meta-analyses dealing with ROS1 and RET depicted a more complex landscape [15, 16]. In the Caucasian cohorts reviewed in the meta-analyses, female gender but not younger age was found to be associated with *ROS1* rearrangement [18, 19]. In our study, the *ROS1*-rearranged patients were younger than the negative patients and *ROS1* rearrangement was not found to be associated with the female gender. Interestingly, our study is the first to report the association of *RET* rearrangement with the female gender in the Caucasian population. On that topic, available data were inconclusive since Michels et al. identified more men and Sarfaty et al. more women among the RET-positive patients in European and Israeli cohorts, respectively [20, 21]. However, as the description of the entire cohort was lacking in both studies, conclusions regarding the association between *RET* rearrangement and gender could not be drawn. Concerning the history of smoking, *ROS1* and *RET* fusions were found to be associated with no or light smoking. Nevertheless, it should be noted that the absolute number of smokers slightly exceeded that of non-smokers. Likewise, Michels et al. also reported smokers among the RET-positive patients in their European cohort [20]. Thus, it would be wise not to exclude smokers from the screening as some diagnostic algorithms suggest [22]. In addition, the stage of the disease at the time of diagnosis of *RET* fusion still remains to be established. Lee et al. reported that *RET* rearrangement mainly affects Asian patients with low-stage disease [17], whereas Michels et al. found more advanced stage disease among the Caucasian patients [20]. Similarly, our findings show a trend for high stage disease at the time of diagnosis. Finally, as reported in Asian studies, *ROS1* and *RET* fusion genes were found in large cell and not otherwise specified carcinoma highlighting that the screening should not be restricted to adenocarcinoma [23, 24].

*ROS1* and *RET* rearrangements have been recently discovered in NSCLC, and the reliability of immunohistochemistry (IHC) in identifying protein overexpression still need to be refined [25, 26]. Two *ROS1*-rearranged samples were negative for ROS1 expression by IHC. The first patient underwent a curative surgery and the second one achieved a complete response with crizotinib, further confirming the aberration of ROS1. IHC staining was also negative for a *ROS1*-amplified sample. Of note, the correlation between overexpression and amplification of ROS1 has been inconsistent [27, 28]. The knowledge gained from studying ALK [29] also questions the consequences of increased copy numbers of *ROS1* or *RET*. *ROS1* CNG was found to be associated with shorter disease-free and overall survival by Jin et al. but not by Clavé et al. [27, 30]. Here, CNG was more frequent for *RET* than for *ROS1* and high levels of CNG (7 to 10 copies) were mainly restricted to *RET*. The level of *RET* CNG was in accordance with the data from Yang et al. [31], but noticeably exceeded 10.9% as reported by Platt et al. [26]. In addition, we revealed a higher frequency of isolated 5′ signals in *RET* compared to *ALK* or *ROS1* FISH profiles. Oncogenic fusions of tyrosine kinases arise from chromosomal breaks, accompanied with or without the loss of adjacent DNA sequences [32, 33]. As most DNA breaks occur proximal to the exons encoding the kinase domain [34], isolated 5′ signals are thought to denote the lack of the kinase domain and are considered negative. One must be aware that FISH probes often hybridize to regions surrounding the genes. Thus, the lack of 3′ signals does not formally rule out the presence of the kinase domain. Recently, Li et al. reported a deletion right next to the kinase domain of *ALK*, which reduced the target region of the probe [35]. The remaining signal could not be detected by FISH, although the kinase domain was present. In addition, currently accumulating data suggest an unexpected complexity in the gene fusion landscape, including alternative breakpoints [36], breakpoints distal to the kinase domain [34, 37] or complex chromosomal rearrangements [32, 38–40]. Thus, caution should be exercised when drawing conclusion from atypical FISH profiles. Whether this observation is clinically relevant is currently unknown. However, if other groups confirm the unexpected frequency, the question whether these patients are eligible to TKIs will arise.

Nearly half of the *ROS1*-rearranged patients were successfully treated with ROS1 TKIs, except one patient who had brain metastases and progressed under crizotinib. This progression could be related to the poor penetration of the blood-brain barrier by crizotinib [41]. In case of relapse, the patients were usually given second- or third-generation TKIs, demonstrating that the *ROS1*-rearranged patients have entered fully into the era of targeted therapies. By contrast, few RET-positive patients received RET TKIs, often in third or subsequent line therapy, and the results have fallen short of expectations. First clinical trials evaluating RET TKIs have shown heterogeneous results with overall response rates ranging from 16 to 54% [42–45]. Of note, these results have been obtained with multi-kinase TKIs that are unlikely to ensure optimal RET inhibition. Fortunately, TKIs with more potent anti-RET activity are in advanced stages of clinical development [46, 47]. Faced with that concern, clinicians frequently prescribed pemetrexed-containing chemotherapy, in agreement with the valuable results that have been reported in fusion gene-driven NSCLC [48]. In addition, although studies have suggested little or no benefit of immune checkpoint inhibitors in oncogene-driven tumors [49], some patients were given nivolumab. A reduced total mutation burden has been proposed as an explanation for the low response rates [50], but confirmatory studies are warranted.

Finally, our study on a large cohort shows that *RET* rearrangement is as frequent as *ROS1* rearrangement in Caucasian NSCLC patients. If available multi-kinase TKIs have provided first clues for efficacy, TKIs designed to more potently target RET are hoped to achieve a better response rate. Given the reported frequencies, the number of patients potentially affected by a rearrangement of *RET* might exceed 20 000 a year, worldwide. Therefore, we recommend the prompt implementation of routine RET testing in non-squamous NSCLC patients, including those with a history of smoking. RNA sequencing, once validated and widely used in pathology laboratories, should considerably aid in such screening and improve the clinical management of RET-positive patients.

## MATERIALS AND METHODS

### Ethics statement

The study was approved by the institutional ethics committee of the Rennes University Hospital.

### Patient and sample selection

The pathology department at Rennes University Hospital is part of a network of hospital molecular genetics platforms that the French National Cancer Institute has been supporting since 2006. Formalin-fixed paraffin-embedded (FFPE) samples of NSCLC were sent at the time of diagnosis for molecular testing. They were collected from a population pool of three or four million inhabitants in western France. Then, the histological subtype was determined by experienced lung pathologists (FLG and DCC). Mutational screening was performed by pyrosequencing (PSQ 96MA, Qiagen, Courtaboeuf, France). Of 3,015 NSCLC samples sent to our pathology department between January 2014 and June 2016, 713 non-squamous NSCLC specimens with wild-type *EGFR/KRAS/HER2/BRAF/PIK3CA/ALK* were prospectively assessed for *ROS1* and *RET* rearrangements by using break-apart FISH assays. Treatment decisions and patient’s care were at the medical oncologists’ discretion. Best response to therapy was assessed using RECIST version 1.1. Data (clinical, pathological and molecular features) were collected centrally and analyzed by an independent statistician.

### Fluorescence *in situ* hybridization

*ROS1* and *RET* FISH assays were performed on 4-μm-thick sections of FFPE tissue blocks using ZytoLight SPEC ROS1 and RET dual color break-apart probes according to the manufacturer’s instructions (ZytoVision, Bremerhaven, Germany). The design of the probes is depicted in Figure 1: the 5′ orange and 3′ green probes hybridized proximal and distal to the kinase domain, respectively. The slides were analyzed by 2 experienced cytogeneticists (FD and FC) by using a fluorescence microscope (Axioskop2, Axio Imager Z2, Zeiss, Göttingen, Germany) and Isis imaging software (Metasystems, Altlussheim, Germany). Per case, at least 100 non-overlapping tumor nuclei were examined. A sample was considered positive for rearrangement if at least 15% of the nuclei showed split signals or isolated 3′ signals. Isolated 5′ signals were thought to result from the deletion of exons encoding the kinase domain and were considered negative. Gene copy number per nucleus was recorded as follows: one copy, two copies, low copy number gain (3 to 6 copies), high copy number gain (7 to 10 copies) and amplification (> 10 copies or innumerable clusters).

### Immunohistochemistry

ROS1 IHC was performed using the D4D6 clone (dilution 1:100; Cell Signaling Technology, Danvers, MA) with an ultrasensitive detection system (OptiView DAB IHC detection and amplification) on a BenchMark XT automated immunostainer (Ventana Medical Systems, Illkirch Graffenstaden, France).

### Mutation testing

Mutational screening was performed by pyrosequencing (PSQ 96MA, Qiagen). Mutations in *EGFR*, *KRAS*, *BRAF* and *PI3KCA* were confirmed by allele-specific PCR (Light cycler^®^ instrument 480 II, Roche molecular diagnostics, Pleasanton, CA). *EGFR* exon 19 deletions were confirmed by the analysis of amplified fragments on polyacrylamide gels, and *EGFR*/*HER2* insertions were confirmed by direct sequencing (3130xl Genetics Analyzer, Life Technologies, Villebon-sur-Yvette, France).

### Statistical analysis

The difference of the average age was assessed with a t test for ROS1 (normality of the distributions, homoscedasticity) and a Welsh test for RET (normality of the distributions, heteroscedasticity). Fisher's exact test for count data was used to investigate odds ratio. Data analysis was conducted using R statistical software.

## Abbreviations

ALK: anaplastic lymphoma kinase
EGFR: epidermal growth factor receptor
FFPE: formalin-fixed paraffin-embedded
FGFR: fibroblast growth factor receptor
FISH: fluorescence *in situ* hybridization
HER2: human epidermal growth factor receptor 2
KRAS: kirsten rat sarcoma viral oncogene homolog
NOS: not otherwise specified
NSCLC: non-small cell lung cancer
PI3KCA: phosphatidylinositol 3-kinase, catalytic subunit alpha
RET: rearranged during transfection
TKI: tyrosine kinase inhibitor
